# Psychometric Evaluation of the New Translated and Culturally Adapted Postpartum Bonding Questionnaire in German-Speaking Women

**DOI:** 10.1007/s10995-024-04029-8

**Published:** 2024-11-27

**Authors:** Pia-Cecilia Steinbrueck, Gabriele Meyer, Gertrud Ayerle

**Affiliations:** 1 Martin Luther University Halle-Wittenberg, Medical Faculty, Institute for Health and Nursing Sciences, Halle, Germany; 2 Albert-Ludwigs-University, Medical Faculty, Institute for Nursing Science, Breisacher Strasse 153, Freiburg, 79110 Germany

**Keywords:** Postpartum bonding questionnaire, Reliability, Validity, Psychometric evaluation, Bonding

## Abstract

**Background:**

This study aimed to evaluate the construct validity and reliability of the newly adapted German version of the Parental Bonding Questionnaire (PBQ) in a group of mothers drawn from the general population, with children aged 12–24 weeks. This assessment followed a thorough linguistic validation, which was conducted through a systematic, multi-step translation process.

**Methods:**

363 women completed the PBQ online 12–24 weeks after delivery. Cronbach’s alpha and exploratory factor analysis (EFA) were used to assess internal consistency reliability and construct validity.

**Results:**

The original 4-factor model could not be confirmed. The new PBQ provides a single factor solution. Ten items were removed from the original 25-item PBQ to produce the abbreviated German PBQ-15, which showed strong internal consistency. The Cronbach’s alpha for this version was 0.86.

**Conclusion:**

While further research is needed to establish diagnostic thresholds and strengthen the construct validity of the shortened version, the German-language 15-item version of the PBQ holds promise as an accessible tool for recognizing bonding issues in a general population of German-speaking women 3–6 months postpartum.

## Significance

Until now, no German-language version of the PBQ has been developed through a structured linguistic and cultural adaptation process, nor has it been utilized in clinical settings by midwives or pediatricians. In this study, the PBQ was translated into German, with adjustments for cultural relevance and linguistic validation using a rigorous multi-step process in line with established guidelines for high-quality questionnaire translation. The resulting German version aligns closely with the original English version in terms of content and structure. This study is the first to evaluate the psychometric properties of the newly adapted German PBQ in a community-based (non-psychiatric) sample of mothers with children aged 12–24 weeks. The newly developed 15-item German-language version of the PBQ has demonstrated validity and reliability as a tool for identifying bonding challenges among German-speaking women in the general population, specifically 3–6 months after childbirth.

## Introduction

The relationship between women and their infants is considered a basic prerequisite for both the child’s cognitive and behavioral development and for the mental health of mothers (Suetsugu et al., 2015). An early parent-child bond, where parents show empathy and attentiveness to their child’s physical and emotional needs, creates a foundation that supports the child’s development of self-esteem and resilience in the future (Bowlby, 1979, 1988; Thompson, 2000; Winston & Chicot, 2016). Conversely, negatively experienced early relationships increase the risk of cognitive, social, and emotional developmental disorders in children (Leclère et al., 2014; Winston & Chicot, 2016).

Although the development of the parent-child relationship takes place throughout life (Bowlby, 1988), there is evidence that there is a sensitive period of relationship development between the child and the primary caregiver in the first six to 24 months of life (Zeanah et al., 2011). Taylor and colleagues (2005) suggest that maternal bonding evolves gradually over the first 12 weeks following childbirth. Research by Wittkowski et al. (2007) indicates that mothers experience a notable increase in positive emotions two to four days postpartum compared to right after delivery. Moehler et al. (2006) observed that vulnerability within the mother-infant relationship, particularly when affected by maternal depression, becomes more pronounced starting in the second week, peaks around the sixth week, and then diminishes after approximately four months. Yoshida et al. (2012) observed that maternal feelings of affection and anger showed a decline at four months postpartum when compared to levels recorded just five days after childbirth. Similarly, Mercer (2004) stated that mothers’ self-reported feelings of love, satisfaction in their role, and positive coping responses to their infant’s irritable behaviors were more pronounced at the four-month mark than at any other time postpartum. Supporting this, Pridham et al. (1999) reported that mothers demonstrated the greatest adaptability in caregiving models around four months after giving birth.

Parental bonding is not an instinctive or immediate process; rather, it is influenced by multiple factors, such as the child’s traits, the psychosocial ressources of the parents, and environmental conditions, among others (Bicking Kinsey & Hupcey, 2013). It is an affective state of the parent in which parental feelings and emotions towards the infant are assumed to be the main indicator of Parent-child bonding (Bicking Kinsey & Hupcey, 2013). Mother-infant bonding is primarily guided by the mother, with her feelings and emotions toward her baby considered the primary indicators of the strength of their bond (Bicking Kinsey & Hupcey, 2013).

Brockington and colleagues (2001) suggest that maternal bonding disorders do not represent a single condition. Instead, they encompass a range of interrelated clinical issues that affect the mother-infant relationship in various pathological ways. These issues may manifest as a lack of maternal affection, irritability, hostility, aggressive tendencies, troubling thoughts, or significant rejection of the child. Such disorders are relatively prevalent among mothers who seek psychiatric care and are observed in 29% of those experiencing postnatal depression (Brockington et al., 2001).

Using a questionnaire for self-disclosure can be a valuable way for women to communicate their emotions (Brockington, 2011). When bonding is a self-perceived phenomenon, questionnaires can serve as cost-effective and suitable tools for evaluating bonding quality and identifying potential bonding disorders (Brockington, 2011). Various self-assessment tools have been developed to assess bonding issues that may arise after childbirth. These include the Maternal Attachment Inventory (Mueller, 1994), Mother-Infant Bonding Scale (Taylor et al., 2005), Parent-to-infant Attachment Questionnaire (Condon & Corkindale, 1998) and the Postnatal Bonding Questionnaire (PBQ) (Brockington et al., 2001). The results of these three tools correlate moderately (van Bussel et al., 2010). The PBQ and its adapted forms have shown robust structural validity, internal consistency, and reliability, as supported by high-quality evidence from numerous studies. This tool is widely utilized in both research and clinical contexts (Brockington et al., 2001; Hornstein et al., 2006; Moehler et al., 2006; Reck et al., 2006; Siu et al., 2010; Suetsugu et al., 2015; van Bussel et al., 2010; Wittkowski et al., 2007; Wittkowski et al., 2020).

Brockington et al. (2001) showed that the four factors of the original version had test-retest reliabilities (Pearson’s r) of 0.95, 0.95, 0.93 and 0.77 in a sample of 30 women. Later studies found that the internal consistency (Cronbach’s α) of the entire questionnaire ranged from 0.76 to 0.87. For individual factors, the “impaired bonding” factor showed consistency between 0.76 and 0.79, the “rejection and anger” factor ranged from 0.63 to 0.75, the “anxiety about care” factor from 0.34 to 0.64, and the “risk of abuse” factor had a lower consistency range of 0.20 to 0.36 (Reck et al., 2006; Suetsugu et al., 2015; van Bussel et al., 2010; Wittkowski et al., 2007). The external validity of the PBQ was confirmed through its correlations with other assessments of mother-child bonding (Reck et al., 2006; Suetsugu et al., 2015; van Bussel et al., 2010; Wittkowski et al., 2007).

However, subsequent validation studies were unable to reproduce the initial four-factor structure (Reck et al., 2006; Wittkowski et al., 2010). A shortened version with 22 items and a three-factor solution was developed by Wittkowski and his colleagues (2010), while Reck and his colleagues (2006) developed a shortened version with 16 items in German. In 2006, Reck and colleagues performed a factor analysis that resulted in a one-factor model and a revised version of the questionnaire containing 16 items, with the majority aligning with the overarching factor, “Impaired Attachment.” Later, in 2010, Wittkowski and colleagues modified the questionnaire by eliminating items from the original Factor 4. This adjustment uncovered a new three-factor structure that somewhat aligned with Brockington’s original three factors. However, the distribution of items within each factor differed slightly.

The PBQ stands out as one of the most frequently translated questionnaires, highlighting its broad applicability and popularity in different settings (Wittkowski et al., 2020). As the PBQ is a self-report questionnaire and is easy to use, it serves as an effective screening tool for midwives, physicians, and other healthcare providers working with young families to identify mothers who may be at risk of bonding difficulties and potential child abuse (van Bussel et al., 2010).

Prior to 2006, there was no German-language questionnaire available to assess mother-infant bonding. The first German version of the Postpartum Bonding Questionnaire (PBQ) was created through a back-translation process and tested on a representative sample of new mothers two weeks postpartum by Reck and colleagues (2006). Although translated, this version of the PBQ had not undergone a formal linguistic and cultural adaptation process and had not been used in clinical practice by midwives or pediatricians. An instrument validated for a specific context may lose its validity in a different time, cultural background or setting (Gjersing et al., 2010). Self-report scales are often subject to biases influenced by cultural factors such as social desirability and response patterns (Yasir, 2016). Translating foreign language instruments requires a structured process to ensure both linguistic and cultural adaptation, aiming for content equivalence (Epstein et al., 2015). Simply translating a questionnaire is inadequate; cultural adaptation is necessary to maintain content consistency and validity. Standardizing translations is essential for valid instruments and reliable data collection (Wild et al., 2005).

In this study, the PBQ was successfully translated into German, with cultural adaptation and linguistic validation achieved through a rigorous multi-stage process based on established guidelines for high-quality questionnaire translation (reference omitted for anonymity). The final German version matches the content and breadth of the original English version and differs from the Reck et al. (2006) translation on 20 of the 25 items (reference omitted for anonymity).

The aim of this study was to assess the psychometric properties of a newly adapted and linguistically validated German version of the PBQ in a community-based sample of women with children aged 12–24 weeks. The timing of the postnatal assessment is selected with the understanding that parents’ views can shift—either positively or negatively—during the initial weeks and months following childbirth. By around the fourth month after a first child’s birth, it is generally assumed that a mother’s self-image as a parent has become more stable, shaped by a relatively realistic understanding of her infant (Busonera et al., 2017). By assessing a community sample, this study aims to capture a range of bonding behaviors and potential difficulties that may not emerge in clinical populations, providing a more comprehensive understanding of the instrument’s utility for early detection of bonding issues. This approach enhances the generalizability of the findings, ensuring the PBQ’s relevance in everyday healthcare settings where women may not present with diagnosed psychiatric disorders but could still benefit from early intervention.

Based on the literature, we hypothesize that the factor structure of the Postnatal Bonding Questionnaire (PBQ) will reflect a multidimensional construct, consisting of three primary factors: impaired bonding, rejection and anger, and anxiety about care. The fourth factor, “risk of abuse,” as originally proposed by Brockington et al. (2001), is expected to show weaker internal consistency and may not emerge distinctly in the current analysis, as seen in later studies (Reck et al., 2006; Wittkowski et al., 2010). Additionally, we expect construct differences in how these factors are represented across different cultural adaptations of the PBQ, with impaired bonding emerging as the most stable factor, as previously observed in German and other translations (Reck et al., 2006; Wittkowski et al., 2010).

## Methods

### Participants

German-speaking women with children aged 12 to 24 weeks were surveyed online for the sample. Recruitment took place from March to August 2022 throughout Germany by sending letters with the access link to the online questionnaire to freelance midwives, registered pediatricians and other people who encounter young families (e.g. baby courses, advice centers and early help). In addition, information was provided about online participation in social networking groups, which are mainly attended by young parents. Women with a full-term single-born baby and who speak German as their mother tongue were invited to participate. Women with multiples, premature babies or sick children were excluded from the survey. The questionnaire was completed anonymously. All participants consented to their data being used in this study.

For this study, at least 300 women were to be recruited for the postnatal survey. There are recommendations available regarding the sample size for the use of exploratory factor analysis (EFA): 100 = poor, 200 = fair, 300 = good, 500 = very good, ≥ 1000 = excellent (Williams et al., 2010). Given the different types of questionnaires, there are no absolute rules for the sample size required to validate a questionnaire (Williams et al., 2010).

### Measuring Instrument

The PBQ, developed by Brockington and colleagues in (2001), is a 25-item questionnaire designed to assess women’s feelings and attitudes towards their infants. It is divided into 4 subscales: impaired bonding” (12 items), “rejection and anger” (7 items), “anxiety of care” (4 items) and “risk of abuse” (2 items). Each item is rated on a 6-point Likert scale from 0 (almost always) to 5 (almost never). Eight items are worded positively and scored in reverse order. Higher values indicate that the parent has a negative attitude towards the child and experiences greater psychological stress when interacting with the child.

A new German translation and cultural adaptation of the PBQ was culturally adapted and linguistically validated by the authors in advance (Steinbrueck et al., 2023) according to the method proposed by the International Society for Pharmaconomics and Outcome Research (ISPOR) Task Force on Translation and Intercultural Adaptation (Wild et al., 2005).

### Data Analysis

Analyses were performed using IBM SPSS software (version 29). Descriptive statistics were computed for the sample characteristics and PBQ items, covering the mean, standard deviation, skewness, and kurtosis. Categorical variables were summarized using absolute frequencies and percentages. To evaluate the model’s adequacy, the Kaiser-Meyer-Olkin (KMO) measure of sampling adequacy, Bartlett’s test for sphericity, and total variance explained were utilized. An exploratory factor analysis (EFA) was conducted to identify any latent relationships among the variables. The exploratory factor analysis (EFA) employed principal axis factoring (PAF) with Varimax rotation and Kaiser normalization, assuming that bonding disorders consist of combined factors associated with mother-child relationship issues. This method is in line with principal axis analysis, emphasizing factor aggregation rather than simple component summation, which is characteristic of principal component analysis.

The selection of the number of significant factors to be retained was based on several criteria: the Scree plot test (focusing on points above the elbow), Kaiser’s criterion (eigenvalues ≥ 1), interpretability, and cumulative variance explained (greater than 40%). Items in the PBQ were retained based on specific factor loading requirements: primary factor loadings had to exceed 0.4, while secondary loadings had to remain below 0.3. Items that did not meet these standards were removed one at a time and the exploratory factor analysis (EFA) was repeated until all remaining items met these conditions.

Reliability was assessed using indices of agreement and consistency. To measure the internal consistency of the PBQ questionnaire subscales and items, Cronbach’s alpha was calculated, with values ≥ 0.7 indicating acceptable reliability. In addition, correlations between items and their respective subscales and total scores were examined to assess how well each item fit within its subscale and the total measure. Item-total correlations of ≥ 0.5 and inter-item correlations of ≥ 0.3 were considered adequate. We expected each item on the scale to assess the same construct, resulting in strong correlations between them.

### Ethical Approval

Participation in this study was entirely voluntary. Informed consent was obtained orally and/or in writing prior to enrolment. The study adhered to the principles of the Declaration of Helsinki and was approved by the Ethics Committee of the Medical of the Martin Luther University Halle-Wittenberg in March 2021.

## Results

### Socio-Demographic Characteristics

A total of 791 women took part in the online survey and 592 completed the questionnaire to the end. After checking exclusion criteria (not female, no child born in the last 12 months), completed questionnaires with monotone missing data of over 20% and removal of careless responses (based on longstring), 560 participants remained. Of these, 363 women had a child aged 12–24 weeks at that time and were included in the analysis. For this sample, item-level missing data were handled under the assumption that it was missing completely at random (MCAR), meaning the missingness was unrelated to the observed or unobserved data. MCAR allows for a more straightforward and unbiased treatment of missing values. Pairwise deletion was employed to handle the missing data, as it maximizes the available data for each analysis by using all cases that have data for the variables being analyzed. This approach helps maintain the sample size for the factor analysis and the estimates of the internal consistency reliability under an MCAR mechanism.

The age of the women was between 21 and 45 years (mean = 32.9 years, SD = 4.1). The average age of the children was 17.3 weeks (SD = 4.1). More than half of the women (60.1%) were first-time mothers. In addition, 75.2% were married or living in a registered civil partnership. In addition, 81.5% had a high school diploma (see Table 1).

**Table 1:** Socio-demographic characteristics of the participating mothers with children between 12–24 weeks of age (*n* = 363)

		Number	Percent	Valid percentages	Cumulative percentages
Age groups	20–29 years	72	19,8	19,9	19,9
	30–39 years	266	73,3	73,7	93,6
	40–49 years	23	6,3	6,4	100,0
	Total	361	99,4	100,0	
School-leaving certificate	Higher degree	295	81,3	81,5	81,5
	Intermediate degree	56	15,4	15,5	97,0
	Other school-leaving qualification	9	2,5	2,5	99,5
	No school leaving qualification	2	0,5	0,5	100,0
	Total	362	99,7	100,0	
Partnership	Divorced	5	1,4	1,4	1,4
	Single	83	22,9	23,1	24,5
	Married	270	74,4	75,2	99,7
	Widowed	1	0,3	0,3	100,0
	Total	359	98,9	100,0	
Number of children	1	218	60,1	60,3	60,3
	2	99	27,3	27,4	87,7
	3	32	8,8	8,9	96,6
	4	8	2,2	2,2	98,8
	5	2	0,6	0,6	99,4
	6	2	0,6	0,6	100,0
	Total	361	99,4	100,0	

The geographical distribution of the participants was as follows: 20.1% from the northern, 25.6% from the eastern, 25.5% from the western and 28.3% from the southern federal states of Germany.

### Factor Structure of the PBQ

The Kaiser-Meyer-Olkin (KMO) measure of sampling adequacy was found to be 0.84, and Bartlett’s test for sphericity was highly significant (χ² = 2693.288, df = 300, *p* < 0.001). These results suggest that the dataset is suitable for exploratory factor analysis (EFA).

The original 4-factor solution of the PBQ, which captured 41.5% of the total variance, was first extracted and rotated. Factor 1, with an eigenvalue of 5.87, explained 23.49% of the variance, while factor 2 (eigenvalue = 1.56) accounted for 6.25%. Factor 3, with an eigenvalue of 0.99, accounted for 3.97% and Factor 4, with an eigenvalue of 0.75, explained 3.0% of the variance. However, the eigenvalues for factors 3 and 4 were too close together, indicating that the original 4-factor structure was not sufficiently supported.

**Fig. 1:**
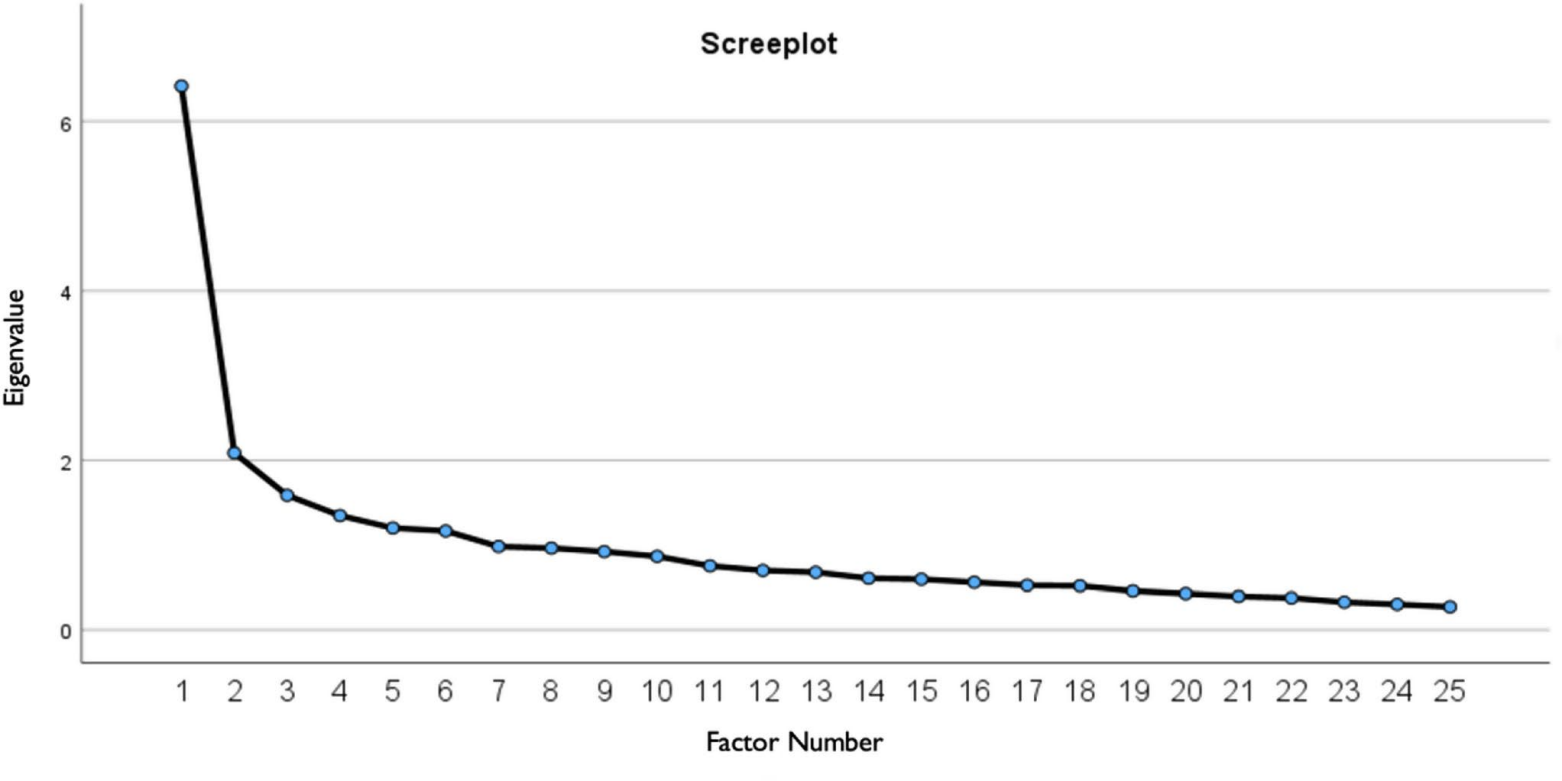
Screeplot for factor analyses of the PBQ

To investigate further, we explored an alternative factor solution for the PBQ. Although two factors had eigenvalues greater than 1, the scree plot (Fig. 1, top) dropped significantly after the first. Therefore, Principal Axis Factoring (PAF) was used to extract a single factor solution. This single factor explained 22.8% of the total variance. We identified factor loadings above 0.40 as sufficiently high for each item, which led us to exclude 10 items that did not meet this criterion. The remaining 15 items, all of which had loadings greater than 0.40, were then subjected to further factor analysis. The final PBQ was determined as a one-factor model with 15 items and accounted for 31.5% of the total variance. The factor loadings suggest that the reliability of each item was adequate, with values ranging from 0.40 to 0.65. Table 2 shows the factor loadings for the 15 items of the PBQ. The one-factor model PBQ-15 comprises 8 items of factor 1, 5 items of factor 2 and two items of factor 3 of the original PBQ. The internal consistency for the German-language PBQ-15 resulted in a Cronbach’s ⍺ of 0.86.

**Table 2:** Factor matrix^a, b^ for the german-language PBQ with 15 items for mothers with 12–24 week old children (*n* = 363)

Items	Factor loading
Q1	0,514
Q2	0,544
Q5	0,429
Q7	0,648
Q8	0,625
Q10	0,403
Q11	0,555
Q12	0,574
Q13	0,642
Q14	0,627
Q17	0,436
Q19	0,582
Q21	0,631
Q23	0,634
Q25	0,483

^a^Age groups child = 12–24 weeks; ^b^ EFA with PAF, one factor extracted

## Discussion

The PBQ was successfully translated into German according to ISPOR criteria, culturally adapted and linguistically validated (Steinbrueck et al., 2023). This study represents the first evaluation of the psychometric properties of the newly developed German-language PBQ.

The original 4-factor structure of the PBQ could not be reproduced. Our findings indicate that, for German-speaking mothers, the PBQ aligns with a single-factor solution. This outcome is consistent with earlier research conducted in German (Reck et al., 2006).

Brockington and colleagues (2001) applied principal component analysis (PCA) to 84 items using varimax rotation for an orthogonal solution in their first English version of the PBQ. Through this analysis they identified 25 items associated with four primary factors. Using PCA, Reck et al. (2006) extracted a one-factor model, Busonera et al. (2017) extracted a three-factor model in an Italian version and Garcia-Esteve et al. (2015) extracted a four-factor model in a Spanish version. The Japanese versions, however, were extracted using PAF and resulted in a one-factor model, a 3-factor model and a 4-factor model (Kaneko & Honjo, 2014; Ohashi et al., 2016; Suetsugu et al., 2015). PAF is more suitable than PCA for identifying latent variables of instrument components. For the present questionnaire items, we assumed that the responses to the items contain measurement error and that the construct “bonding disorder” captured by the items could be a latent variable. As a result, we decided to use PAF. Given the notably high eigenvalue of the first factor, we developed a condensed version with 15 PBQ items, structured around a single factor.

Although 10 items were excluded from the original 25-item PBQ, the shortened German PBQ-15 questionnaire showed good internal consistency, with a Cronbach’s alpha for the questionnaire of 0.86. This result is well above the traditional threshold of 0.7 and is comparable to other studies on the translation of the PBQ, which showed a Cronbach’s alpha score between 0.81 and 0.91 (Brockington et al., 2001; Garcia-Esteve et al., 2015; Ohashi et al., 2016; Reck et al., 2006; Suetsugu et al., 2015), indicating a strong interrelationship between the items in the instrument. Both the average inter-item correlation and the average item-total correlation were high, reflecting a solid internal reliability of the instrument.

A possible reason for these different findings may be differences in the study populations. Previous studies have examined the benefits of PBQs specifically for individuals with depression and psychosis in psychiatric settings (Moehler et al., 2006; Wittkowski et al., 2010). Studies of mother-child bonding in the general population are limited, and much of the research on mother-child bonding has focused on clinical groups. Unlike clinical samples, where bonding difficulties may be more severe or influenced by underlying psychiatric conditions, a community-based, non-clinical population reflects a broader range of maternal experiences, from typical bonding to mild difficulties. This difference is significant because it suggests that the PBQ may function differently across populations, with non-clinical samples likely exhibiting subtler bonding issues that might not be as pronounced as in clinical settings.

In non-clinical populations, the need for a practical, user-friendly screening tool like the shortened German PBQ-15 becomes evident. Given our results and the results of previous studies (Kaneko & Honjo, 2014; Reck et al., 2006; Wittkowski et al., 2007), the shortened German PBQ-15 is more suitable for detecting bonding issues in a general population where severe bonding disorders are less common. It allows for efficient and early identification of at-risk mothers, which can be beneficial for early intervention without overburdening respondents with lengthy questionnaires. The focus on a non-clinical sample highlights the adaptability of the PBQ for everyday healthcare settings, extending its applicability beyond psychiatric units and into routine screening in the general maternal population.

This study has several limitations. Self-reported measures are naturally prone to social desirability bias. Online assessments significantly reduce the effects of social desirability, as previous authors have suggested (Joinson, 1999). It should also be noted that in the specific case of mother-child bonding, social desirability has been described as a risk factor (Tsuchida et al., 2019). In addition, public acknowledgement of difficulties with mother-infant bonding tends to be associated with fear of stigmatization, so the anonymity offered by the internet may give mothers more confidence to acknowledge this (Lasheras et al., 2022). Despite the anonymity of online participation, which should be a guarantee of confidentiality, the explanation of the aims of the study could lead to bias because participants want to please the interview team (Hogg & Vaughan, 1998). Despite the bias that can distort interpretation, self-report instruments are less expensive and less labor intensive to use (Streiner et al., 2015). In addition, these instruments provide insight into parents’ personal perspectives on their relationships with their children, which is valuable in research contexts (Condon & Corkindale, 1998). In clinical practice, reliable and valid measures that are easy and efficient to administer can help to identify challenges within the parent-child relationship and to assess progress over time (Brockington et al., 2001).

It must also be acknowledged that further construct validity testing is essential before thresholds can be established, particularly given the reduction in items from 25 to 15. Thresholds require a thorough understanding of the instrument’s sensitivity and specificity, which will only be achievable once construct, convergent, and discriminant validity are well-established. While the shortened PBQ-15 aims to provide a more practical and efficient tool for screening maternal bonding difficulties, current evidence does not yet fully support its construct validity or comprehensiveness in assessing the full range of maternal or parental bonding impairments. To address this, future research should focus on additional validation studies, including the assessment of both convergent and discriminant validity. These studies would help to clarify how well the PBQ-15 measures bonding difficulties compared with other established instruments, and whether it can accurately distinguish between bonding disorders and other psychological conditions.

## Conclusion

In conclusion, the German-language PBQ-15 is not intended to serve as a psychiatric diagnostic tool but rather as a practical, cost-effective screening instrument for use by midwives, pediatricians, and health visitors. Its primary purpose is to help healthcare professionals identify early signs of maternal bonding difficulties, enabling timely intervention. While further research is needed to establish diagnostic thresholds and strengthen the construct validity of the shortened version, the German-language PBQ-15 holds promise as an accessible tool for recognizing bonding issues in non-clinical settings.

## Data Availability

The datasets used and/or analyzed during the current study are available from the corresponding author on reasonable request.

## Abbreviations

e.g.: For example
EFA: Exploratory factor analysis
ISPOR: International Society for Pharmaconomics and Outcome Research
KMO: Kaiser-Meyer Olkin
PBQ: Postpartum Bonding Questionnaire
PAF: Principal axis analysis
PCA: Principal component analysis
